# Idebenone Alleviates Neuroinflammation and Modulates Microglial Polarization in LPS-Stimulated BV2 Cells and MPTP-Induced Parkinson’s Disease Mice

**DOI:** 10.3389/fncel.2018.00529

**Published:** 2019-01-09

**Authors:** Aijuan Yan, Zhihua Liu, Lu Song, Xijin Wang, Yu Zhang, Na Wu, Jingya Lin, Ying Liu, Zhenguo Liu

**Affiliations:** Department of Neurology, Xinhua Hospital Affiliated to Shanghai Jiao Tong University School of Medicine, Shanghai, China

**Keywords:** idebenone, neuroinflammation, microglial polarization, Parkinson’s disease, BV2 cells

## Abstract

**Background:** Idebenone is an antioxidant and a coenzyme Q10 analog that has been used to treat neurodegeneration disease. Some studies show idebenone exerts anti-inflammatory effects. However, whether idebenone can be used to reduce the neuroinflammation in Parkinson’s disease (PD) has been little studied.

**Methods:** The study investigated the potential anti-inflammatory effects of idebenone *in vitro* and *in vivo*, using cell models of Lipopolysaccharide (LPS)-simulated BV2 cells and animal models of 1-methyl-4-phenyl-1,2,3,6-tetrahydropyridine (MPTP)-induced PD with or without idebenone. To verify how idebenone exerts its effects on the BV2 cell activation and PD model, we performed the mechanistic studies *in vitro* and *in vivo*.

**Results:** *In vitro* study showed that pretreatment with idebenone could attenuate the production of pro-inflammatory factors in LPS-stimulated BV2 cells and promoted a phenotypic switch from the M1 state to the M2 state. Mechanistically, idebenone reduced the activation of the MAPK and NF-κB signaling pathway upon LPS stimulation. Furthermore, *in vivo* experiments confirmed that pretreatment with idebenone could ameliorate MPTP-induced neurodegeneration and modulate microglia phenotypes through inhibition of the MAPK and NF-κB signaling pathway in the SN.

**Conclusion:** These results suggest that idebenone ameliorates the neurological deficits related to PD and this effect is partly mediated by inhibiting the neuroinflammation and modulating microglia phenotypes.

## Introduction

Parkinson’s disease (PD) is a neurodegenerative disease that is caused by progressive loss of dopaminergic neurons, Lewy body formation, and mitochondrial dysfunction (Dauer and Przedborski, 2003). In addition to these pathophysiological mechanisms, there is some evidence from previous studies suggest that the neuroinflammatory response related to microglia cell activation participate in the progression of dopaminergic neuron cell death (Czlonkowska et al., 2002; Whitton, 2007; Brochard et al., 2009).

Microglia cells are the main glial cell type that participate in the neuroinflammatory response in the central nervous system (CNS) (Saijo and Glass, 2011; Lu et al., 2018). Under pathological conditions, microglia cells in the brain undergo changes in morphology to adopt an activated state in order to respond to the toxic environment (Song and Suk, 2017). One of the characteristics of microglia cell is the capacity to adopt two different states in response to the toxic substance they encounter. Microglial activation can be classified into two major phenotypes defined as classically M1 microglia or alternatively M2 microglia (Colton, 2009; Han et al., 2018). The M1 phenotype is associated with the release of proinflammatory factors, whereas M2 microglia execute an anti-inflammatory effect and contribute to regeneration and neuroprotection (Saijo et al., 2009; Colton and Wilcock, 2010). M1 microglia release proinflammatory factors such as interleukin-6 (IL-6), IL-1β, tumor necrosis factor-α (TNF-α) to recruit additional inflammatory cells (Giaume et al., 2007; Colton and Wilcock, 2010). M2 microglia produce anti-inflammatory cytokine including IL-4 and IL-10 and promote wound healing and tissue repair (Liu et al., 2006; Yan et al., 2016). The M1 and M2 activation state are critical in neuroinflammation-related neurodegeneration diseases including PD.

Idebenone (2,3-dimethoxy-5-methyl-6-(10-hydroxydecyl)-1,4-benzoquinone), a synthetic analog of coenzyme Q10, is an important antioxidant for cell membrane and an elementary constituent of the ATP-producing mitochondrial electron transport chain (Muscoli et al., 2002). One of the most important features of idebenone is its antioxidant capacity (Jaber and Polster, 2015). Beneficial effects of idebenone have been studied in the treatment of some neurodegeneration diseases, including Parkinson’s disease (Horvath et al., 2003), Alzheimer’s disease (Weyer et al., 1997), and dementia (Voronkova and Meleshkov, 2009). In addition, idebenone is also used in the treatment of Friedreich’s Ataxia, which is a progressive neurodegenerative disease associated with cardiomyopathy and other features (Parkinson et al., 2013). Apart from its antioxidant properties, the study from Civenni et al. (1999) reported that idebenone exerted anti-inflammatory effects and behaved similar to indomethacin and piroxicam—two typical anti-inflammatory agents.

Taking these factors into consideration, we hypothesize that idebenone may have an inhibitory effect on neuro-inflammatory processes and microglial activation in PD. To test this hypothesis, we will investigate the anti-neuroinflammation and microglia modulating effects of idebenone in the BV2 microglial cell line and examine idebenone’s efficacy on 1-methyl-4-phenyl-1,2,3,6-tetrahydropyridine (MPTP)-induced neurodegeneration and neuroinflammatory response in PD mice.

## Materials and Methods

### Reagents

Idebenone was purchased from Qilu Pharmaceutical Co., Ltd., MPTP, dopamine and 3,4-dihydroxyphenylacetic acid (DOPAC) standards, dimethyl sulfoxide (DMSO), and Lipopolysaccharide (LPS) were purchased from Sigma-Aldrich (St. Louis, MO, United States).

### Animals

Male C57BL/6 mice (mean weight = 22–25 g; mean age = 10–12 weeks) were purchased from Shanghai SLAG Laboratory Animal Corporation (Shanghai, China). The mice were individually housed at a temperature of 22 ± 1°C and relative humidity 50–60% under a 12-h/12-h light–dark cycle, with free access to food and water. Animals received four intraperitoneal injection of MPTP (20 mg/kg) at 2 h intervals, whereas control mice were treated with the same volume of saline. Idebenone was resuspended in corn oil and applied daily by oral gavage. 100 mg/kg idebenone or vehicle (corn oil) was administered daily starting at day 7 before MPTP administration (Abdel Baky et al., 2010). These mice were sacrificed at 3, and 7 days after MPTP treatment.

This study was carried out according to the guidelines of the National Institutes of Health and was approved by the Ethics Committee of Xin Hua Hospital affiliated to Shanghai Jiao Tong University School of Medicine.

### Behavioral Tests

#### Open Field Test

Motor behavior was analyzed in an open-field test after 3 and 7 days of administration of MPTP. Open-field test is an important method for assessing spontaneous locomotor activity in mice model of PD (Yan et al., 2018). Mice were placed into the center of the case, and their behaviors were observed. The total distance and the number of gridline crossings were measured by an observer who was seated approximately 1.0 m away. The parameters were analyzed by the SuperMaze V2.0 software (Shanghai Xin Ruan, China) over a 5-min period: the total distance traveled and the number of gridline crossings.

#### Pole Test

The pole test is an available method to evaluate bradykinesia in PD mice model. We performed the pole test at 3 and 7 days after the last MPTP injection according to a previously described protocol (Wu et al., 2012). In brief, a rod with a length of 55 cm and a diameter of 10 mm was used to make the pole. The mice were put on the top of the pole. Time taken by the animal to turn around was recorded as the T-turn (time to turn). The test was carried out three times for each mouse, and the mean time was calculated for statistical analysis.

### Cell Culture

The BV2 microglial cell line was purchased from China Center for Type Culture Collection^1^. The SH-SY5Y cells were presented by Institute of Neurology, Rui Jin Hospital (Shanghai, China). The BV2 microglial cells and SH-SY5Y cells were cultured in Dulbecco’s-modified Eagle’s medium (DMEM) (Gibco; Thermo Fisher Scientific, Inc.) supplemented with 10% fetal bovine serum (FBS) (Gibco; Thermo Fisher Scientific, Inc.) and 1% penicillin/streptomycin at 37°C with an atmosphere of 5% CO_2_. Idebenone was dissolved in DMSO as a 1 M stock solution, and dilutions were made in DMEM. The final concentration of DMSO in the DMEM was less than 0.05% which had no effect on cell growth.

### Cell Viability Assay

The BV2 microglial cells were seeded in 96-well plates (4.0 × 10^3^ cells/ml) and treated with idebenone concentrations (1, 2.5, 5, 7.5, 10, or 20 μM) for 24 h. Cell viability was examined by a CCK-8 assay kit (CCK-8; Dojindo, Tokyo, Japan) according to the manufacturer’s instructions (Yan et al., 2017).

BV2 cells were seeded in 6-well plates (4.0 × 10^5^ cells/ml) and treated with 5 μM idebenone for 2 h, followed by incubation with 100 ng/ml LPS for 24 h. Then, conditioned media of the BV2 cells was collected and applied to SH-SY5Y cells that has been seeded in 96-well plates. CCK-8 assay kit was used to assess the changes in SH-SY5Y cell viability after 24 h. Morphological change in the SH-SY5Y cells were examined by phase-contrast microscopy.

### NO Measurement

The BV2 microglial cells were seeded in 24-well plates (1.0 × 10^5^ cells/ml) and treated with various idebenone concentrations (1, 2.5, 5, 7.5, 10, or 20 μM) for 2 h, followed by 100 ng/ml LPS for 24 h. After 24 h, culture supernatant was collected, and the NO concentration in the supernatant was measured by an NO assay kit (Beyotime Institute of Biotechnology, Inc., Shanghai, China) according to the manufacturer’s instructions (Yang et al., 2016).

### Immunohistochemistry

Immunohistochemical staining of brain sections was carried out as previously described (Luster et al., 2005). Briefly, mice were transcardially perfused with PBS, followed by 4% paraformaldehyde (PFA) in 0.1 M PBS. The brains were removed and immersed for 24 h in 4% PFA, and then cryoprotected in a 30% sucrose solution in PBS (pH 7.4). Subsequently, serial coronal sections (cut thickness: 20 μm) were then prepared using a microtome. These brain sections were immersed with 0.3% H_2_O_2_ for 30 min and then were rinsed with PBS containing 0.3% Triton X-100. The coronal sections were immersed with serum for 1 h and then incubated overnight with a rabbit polyclonal anti-TH (cat. no. 41528, 1:500, Abcam) at 4°C. Staining was carried out using the ABC method (Vector Laboratories), with 3,3′-diaminobenzidine (DAB) as the peroxidase substrate. TH-positive DA neurons from the substantia nigra (SN) region were examined by light microscopy. Stereological counting of TH-positive neurons was used the method described in previous studies (Hunot et al., 2004; Brochard et al., 2009).

Brain sections were fixed with 4% PFA for 15 min and then rinsed with PBS containing 0.3% Triton X-100. Sections were blocked for 60 min in 10% donkey serum and incubated with primary antibody at 4°C overnight. Primary antibodies used were rabbit anti-Iba-1 (cat. no. 178847, 1:200 dilution, Abcam); goat anti-Arg-1 (cat. no. 18351, 1:100 dilution, Santa Cruz Biotechnology); mouse anti-CD16/32 antibody (cat. no. 553141, 1:200 dilution, BD Pharmingen, San Jose, CA, United States). After washing three times with PBS, brain sections were incubated with corresponding donkey anti-rabbit, donkey anti-goat, or donkey anti-mouse secondary antibodies conjugated to Alexa Fluor-488 and Alexa Fluor-594 fluorochrome (1:500 dilution, Life Technologies). 4,6-diamidino-2-phenylindole (DAPI) (1:1000 dilution, Beyotime Institute of Biotechnology, China) was used to stain the nuclei. The brain sections were examined by a microscope (Leica, Solms, Germany).

BV2 microglial cells were fixed with 4% PFA and stained overnight with rabbit anti-NF-κB antibody (cat. no. 32536, 1:300, Abcam) at 4°C, followed by incubation with the fluorescent conjugated secondary antibodies.

### High-Performance Liquid Chromatography (HPLC) Analysis

The levels of dopamine and its metabolites (DOPAC) in the striatum were measured using an HPLC apparatus (Yu et al., 2017). Briefly, the striatum was weighed and quickly removed on ice. Striatum was homogenized in perchloric acid (0.1 mol/L). After lysis, samples were centrifuged at 10,000 *g* (4°C) for 10 min and the collected supernatants were then assayed for dopamine and DOPAC content through HPLC. The content of each neurotransmitter was expressed as pg/mg equivalent striatal tissue.

### Reverse Transcription-Quantitative Polymerase Chain Reaction (RT-qPCR)

BV2 cells, seeded in 12-well plates, were pretreated with 5 μM idebenone for 2 h, followed by treatment with 100 ng/ml LPS for 12 h. Total RNA was extracted from BV2 microglial cells with Trizol reagent (Takara Bio, Inc., Otsu, Japan) and was reverse transcribed to cDNA using the PrimeScript RT Reagent kit (Takara Bio, Inc., Otsu, Japan), as previously described (Yan et al., 2017). RT-qPCR was carried out using an SYBR Green kit (Takara Bio, Inc., Otsu, Japan) with the following conditions: Denaturation at 95°C for 10 s, followed by 40 cycles at 95°C for 5 s and 60°C for 30 s. The data were analyzed using the comparative threshold cycle (Ct) method. The outcome was expressed as fold-difference normalized to the ribosomal phosphoprotein P0 (Rplp0). RT-qPCR was detected using an ABI PRISM 7500 Sequence Detection system (Thermo Fisher Scientific, Inc.). The primers used in RT-qPCR were as follows:

IL-6 (F: TAGTCCTTCCTACCCCAATTTCC, R: TTGGTCCTTAGCCACTCCTTC); IL-1β (F: GCAACTGTTCCTGAACTCAACT, R: ATCTTTTGGGGTCCGTCAACT); TNF-α (F: CCCTCACACTCAGATCATCTTCT, R: GCTACGACGTGGGCTACAG); iNOS (F: ATGTCCGAAGCAAACATCAC, R: TAATGTCCAGGAAGTAGGTG); CD 16 (F:TTTGGACACCCAGATGTTTCAG, R: GTCTTCCTTGAGCACCTGGATC);CD32 (F: AATCCTGCCGTTCCTACTGATC, R:GTGTCACCGTGTCTTCCTTGAG); CD86 (F: TTGTGTGTGTTCTGGAAACGGAG, R: AACTTAGAGGCTGTGTTGCTGGG); Arg-1 (F: GAACACGGCAGTGGCTTTAAC, R: TGCTTAGCTCTGTCTGCTTTGC); CD206 (F: TCTTTGCCTTTCCCAGTCTCC, R: TGACACCCAGCGGAATTTC);YM (F: CAGGGTAATGAGTGGGTTGG, R: CACGG CACCTCCTAAATTGT); Rplp0 (F: AGATTCGGGATATGCTGTTGGC, R: TCGG GTCCTAGACCAGTGTTC).

### Western Blotting

Proteins were extracted from the BV2 cells and brain by homogenization in standard lysis buffer. The concentration of protein was measured using a BCA kit (Beyotime Institute of Biotechnology, Inc., Shanghai, China) according to the manufacturer’s instructions (Cai et al., 2018). 40 μg of protein was separated by 10% SDS-PAGE and then transferred into nitrocellulose membranes (300 mA for 60 min). The membranes were blocked with 3% bovine serum albumin (BSA) for 1 h and immersed overnight in primary antibodies at 4°C. The primary antibodies used were as follows: p-ERK (cat. no. #4370), ERK (cat. no. #4695), p-p38 (cat. no. #4511), p-38 (cat. no. #8690), p-JNK (cat. no. #4668), JNK (cat. no. #9252), p-NF-κB (cat. no. #3033), NF-κB (cat. no. #8242) (1:1,000; Cell Signaling Technology, Inc., Danvers, MA, United States), a rabbit polyclonal anti-TH (cat. no. 41528, 1:500, Abcam). The membranes were washed three times with Tris-buffered saline and Tween 20 (Beyotime Institute of Biotechnology, Inc., Shanghai, China) and incubated with corresponding anti-rabbit Horseradish Peroxidase (HRP) IgGs (cat. no. #7074 diluted 1:1000, Santa Cruz Biotechnology, CA, United States) secondary antibody for 1 h. The bands were visualized by chemiluminescence (Thermo Scientific Inc.) and results were measured by a ChemiDoc^TM^ XRS+ imaging system (Bio-Rad Laboratories, Inc., Hercules, CA, United States).

### Statistical Analysis

All data were analyzed by GraphPad Prism software, v6.0 (GraphPad Software, Inc., La Jolla, CA, United States). Comparison between the two groups was assessed with an unpaired *t*-test, while comparison among several groups was evaluated using one-way ANOVA followed by a Tukey’s multiple comparisons test. Data were expressed as the mean ± standard error of the mean (SEM). *p*-values < 0.05 was considered statistically significant.

## Results

### Idebenone Reduced NO Release and Expression of Proinflammatory Cytokines in LPS-Activated BV2 Cells

**FIGURE 1:**
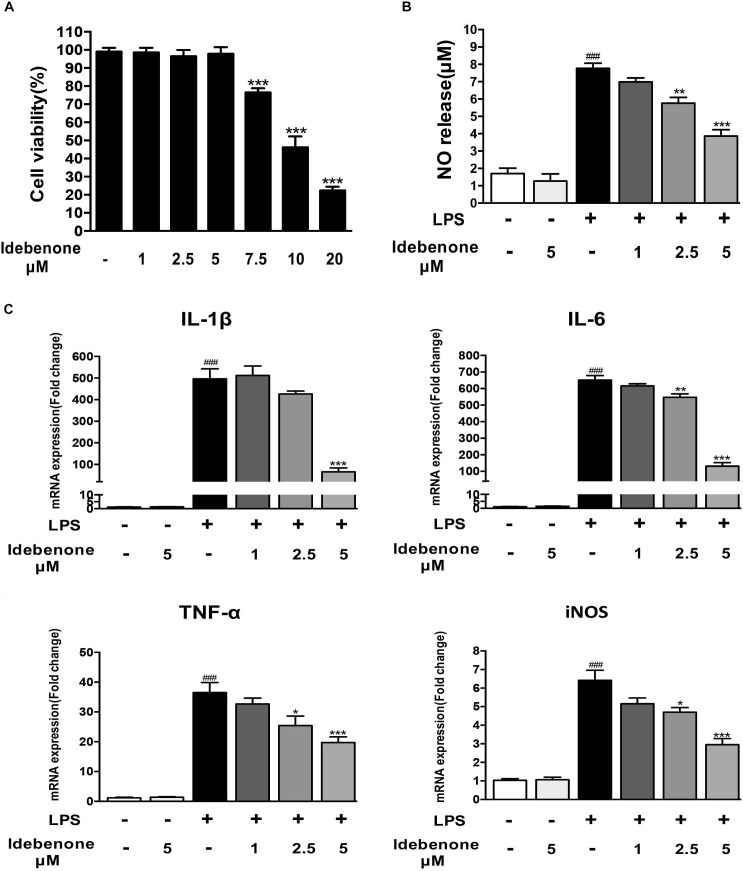
Idebenone reduced LPS-induced NO release and expression of proinflammatory cytokines in BV2 cells. **(A)** Effects of different concentration of idebenone on BV2 microglial cell viability. ^∗∗∗^*p* < 0.001, different concentration of idebenone vs. control. **(B)** The NO content of the supernatant was analyzed using NO assay kit. **(C)** The mRNA expression levels of IL-1β, IL-6, TNF-α, and iNOS were measured by RT-qPCR in different group. ^∗^*p* < 0.05, ^∗∗^*p* < 0.01, ^∗∗∗^*p* < 0.001: idebenone + LPS vs. LPS; ^###^*p* < 0.001: LPS vs. vehicle.

To detect the potential cytotoxicity of idebenone, we analyzed the dose-dependent effects of idebenone (1, 2.5, 5, 7.5, 10, and 20 μM) on the survival of BV2 microglial cells. The result demonstrated that idebenone was not cytotoxic to BV2 microglial cells at 1, 2.5, or 5 μM (Figure 1A). Activated microglia release various proinflammatory cytokines, NO and superoxide (Shao et al., 2013). We then investigated the effects of idebenone on LPS-activated BV2 cells. Our data showed that idebenone (1, 2.5, and 5 μM) dose-dependently decreased the LPS-stimulated production of NO (*p* < 0.01; Figure 1B) and suppressed the mRNA expression of IL-6, IL-1β, TNF-α, and iNOS in LPS-stimulated BV2 cells (*p* < 0.05; Figure 1C). As shown in Figure 1, the most inhibition was observed at the concentration of 5 μM, and we selected 5 μM idebenone in the following experiments.

### Idebenone Suppressed LPS-Stimulated Microglial M1 Polarization and Promoted M2 Polarization in BV2 Cells

**FIGURE 2:**
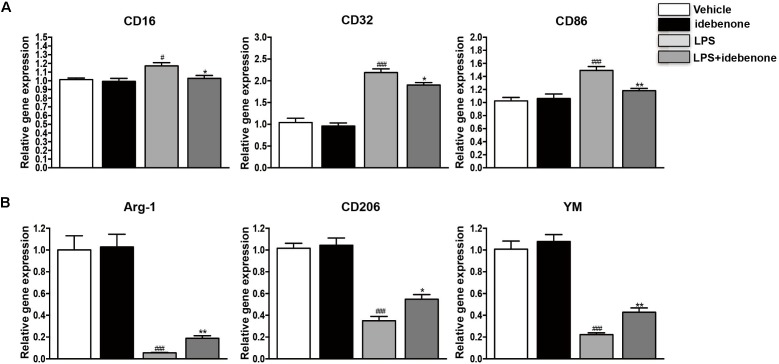
Idebenone reduced the expression of M1 markers and promoted the expression of M2 markers in LPS-activated BV2 cells. **(A)** The expression of M1 markers (CD16, CD32, and CD86) as determined by RT-qPCR in the different groups. **(B)** The expression of M2 microglial markers (Arg-1, CD206, and YM) as determined by RT-qPCR in the different groups. ^∗^*p* < 0.05, ^∗∗^*p* < 0.01: idebenone + LPS vs. LPS; ^#^*p* < 0.05, ^###^*p* < 0.001: LPS vs. vehicle.

Lipopolysaccharide is regard as a classically M1 microglia inducer, which causes M1 phenotype to express pro-inflammatory cytokines (Yang et al., 2017). In our study, we showed that the expression of CD16, CD32, and CD86 (M1 markers) was increased in LPS-activated BV2 cells. Idebenone had little impact on microglia polarization in resting state, while it could suppress the expression of M1 markers (*p* < 0.05; Figure 2A) and promote activated microglia to alternatively M2 phenotype in conditions of inflammatory stimulation, as demonstrated by the increased expression of Arg-1, CD206, and YM (M2 markers) (*p* < 0.05; Figure 2B).

### Conditioned Medium From Activated BV2 Cells Pretreated With Idebenone Could Increase SH-SY5Y Cells Viability

**FIGURE 3:**
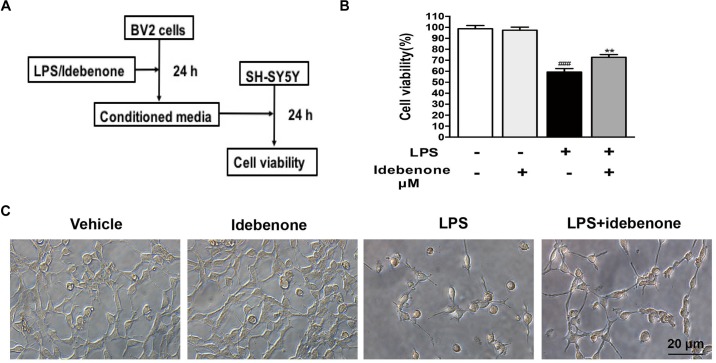
Conditioned medium from idebenone in the pretreated group could increase SH-SY5Y cell viability. **(A)** The experimental design and procedure. **(B)**. The effects of mediated conditioned medium from the different groups on the SH-SY5Y cells were assessed by CCK-8 assay kit. **(C)** The effects of mediated conditioned medium from the different groups on SH-SY5Y cells were observed by microscopy. Scale bar = 20 μm. ^∗∗^*p* < 0.01: idebenone + LPS vs. LPS; ^###^*p* < 0.001: LPS vs. vehicle.

Conditioned medium from LPS-stimulated BV2 cells should contain proinflammatory cytokines and other toxic molecules (Yang et al., 2016). We designed the following experiment to test such impacts on human neuroblastoma SH-SY5Y (Figure 3A). SH-SY5Y, a neuronal cell line, was used to mimic neurons. BV2 microglial cells were treated with 100 ng/ml LPS for 24 h with or without idebenone (5 μM) pretreatment, and then the conditioned medium from BV2 microglial cells was collected. SH-SY5Y cells were incubated with the conditioned medium and cells viability was analyzed by CCK8 assay kit after 24 h. Conditioned medium from LPS-stimulated BV2 cells reduced SH-SY5Y cells viability, and caused morphological changes, while pretreatment with idebenone partially blocked the effect of LPS (Figures 3B,C). These results indicated that LPS could promote production of pro-inflammatory cytokines, which could explain the reduction of SH-SY5Y cells survival in the conditioned medium from activated BV2 cells. Pretreatment with idebenone could reverse the process of inflammation. To eliminate the possibility that reagents in conditioned medium may affect SH-SY5Y cells survival, idebenone and LPS were directly added to SH-SY5Y cells for 24 h. Results suggested that there was no statistically significance among different groups (Supplementary Figure S1).

### Idebenone Inhibited LPS-Induced Activation of Mitogen Activated Protein Kinases (MAPK) and NF-κB

**FIGURE 4:**
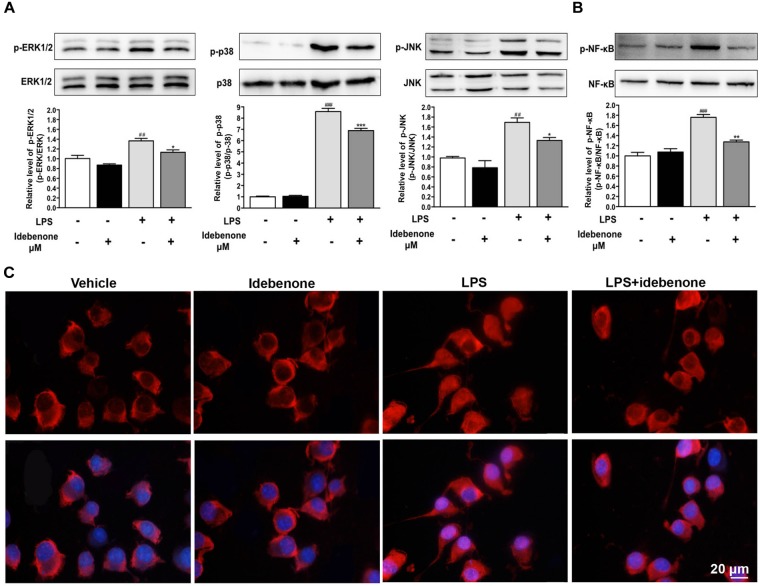
Idebenone inhibited LPS-induced activation of MAPKs and NF-κB. **(A)** Expression levels of p-ERK, ERK, p-p38, P-38, p-JNK, and JNK were analyzed by western blotting. **(B)** Expression levels of p-NF-κB and NF-κB were analyzed by western blotting. **(C)** Idebenone inhibited LPS-caused translocation of p65 from cytosol to nucleus. BV2 microglial cells were immunostained with anti-NF-κB p65 antibodies and Alexa Fluor 594-conjugated secondary antibodies. Scale bar = 20 μm. Values are presented as the mean ± standard error. ^∗^*p* < 0.05, ^∗∗^*p* < 0.01, ^∗∗∗^*p* < 0.001: idebenone + LPS vs. LPS; ^##^*p* < 0.01, ^###^*p* < 0.001: LPS vs. vehicle.

Lipopolysaccharide, an inducer of M1 microglia (Fenn et al., 2012), especially acts on Toll-like receptors (TLRs) of cells, which can stimulate the activation of NF-κB/MAPK pathway. MAPKs are important regulators in the process of LPS-activated BV2 microglial cells (Yan et al., 2017). To detect the potential mechanisms of the idebenone-mediated reduction of inflammatory factors expression and modulation of microglial polarization, BV2 microglial cells were pretreated with or without idebenone for 2 h and then treated with 100 ng/ml LPS for 30 min. LPS induced an increase in the phosphorylation of ERK, p38, and JNK, which was inhibited by idebenone pretreatment (*p* < 0.05; Figure 4A).

The NF-κB signaling pathway is also a significant mediator in LPS-activated BV2 microglial cells (Min et al., 2014). Thus, NF-κB pathway was evaluated as a potential mechanistic target for idebenone during inflammatory response. In the following study, the effect of idebenone on LPS-stimulated NF-κB activation was observed. The data showed that pretreatment with idebenone did not change the protein expression of NF-κB, but it inhibited LPS-induced expression of p-NF-κB in BV2 microglial cells (*p* < 0.01; Figure 4B). The result was confirmed by immunofluorescence study of NF-κB translocation from the cytosol to the nucleus in BV2 microglial cells (Figure 4C).

### Idebenone Treatment Protected Against MPTP-Induced Motor Dysfunction and Reduced the Neuronal Death in the SN and in the Striatum of PD Mice

**FIGURE 5:**
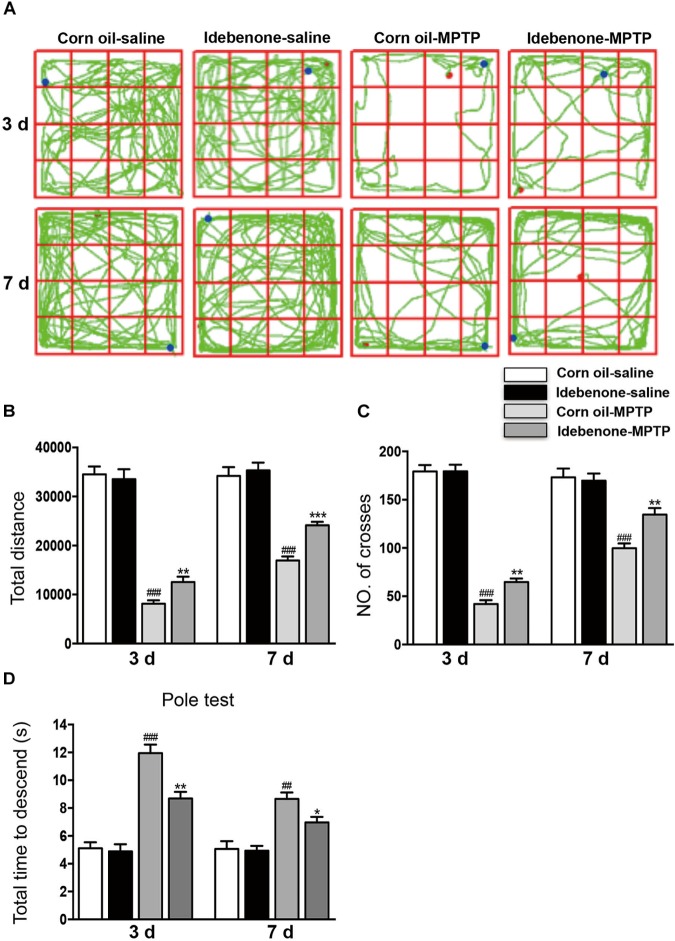
Idebenone treatments ameliorated motor function in MPTP-treated mice. **(A)** Movement paths in the different experimental groups. **(B)** Total distance traveled in different experimental groups. **(C)** The number of squares crossed by mice. **(D)** For the pole test, the time for every mouse to reach the bottom of the pole was recorded and analyzed. Values are presented as the mean ± SEM. ^∗^*p* < 0.05, ^∗∗^*p* < 0.01, ^∗∗∗^*p* < 0.001: idebenone-MPTP vs. corn oil-MPTP; ^##^*p* < 0.01, ^###^*p* < 0.001: corn oil-MPTP vs. corn oil-saline.

We sought to identify whether idebenone treatment affected MPTP-induced dopaminergic neuronal death and neurobehavioral deficits. According to previous studies, idebenone was treated at a dose of 100 mg/kg body weight (Abdel Baky et al., 2010; Fiebiger et al., 2013). Mice received idebenone showed no visible disorders such as reduced appetite, infection, or inhibition of motor activity. The open field test and pole test are useful methods for evaluating the motor dysfunction caused by PD. For open field test, we mapped the mice’s paths on the 3rd and 7th days, which were recorded as the total distance mice traveled in 5 min. MPTP-treated mice that were pretreated with corn oil moved around the arena very little, compared with MPTP-treated mice that received idebenone (3 days: *p* < 0.01; 7 days: *p* < 0.001; Figures 5A,B). Corn oil-MPTP-treated mice demonstrated decreased locomotor activity, crossing fewer lines than corn oil-saline-treated mice (3 days: *p* < 0.001; 7 days: *p* < 0.001; Figures 5A,C). Idebenone-MPTP treated mice crossed more lines than corn oil-MPTP-treated mice (3 days: *p* < 0.01; 7 days: *p* < 0.01; Figures 5A,C). For the pole test, the time to descend for the corn oil-MPTP group was markedly prolonged compared with the corn oil-saline group (3 days: *p* < 0.001; 7 days: *p* < 0.01; Figure 5D). However, the time to descend of the idebenone-MPTP group was shortened to 8.69 ± 0.64 s (3 days, *p* < 0.01; Figure 5D), and 6.97 ± 0.54 s (7 days, *p* < 0.05; Figure 5D) compared with the corn oil-MPTP group.

**FIGURE 6:**
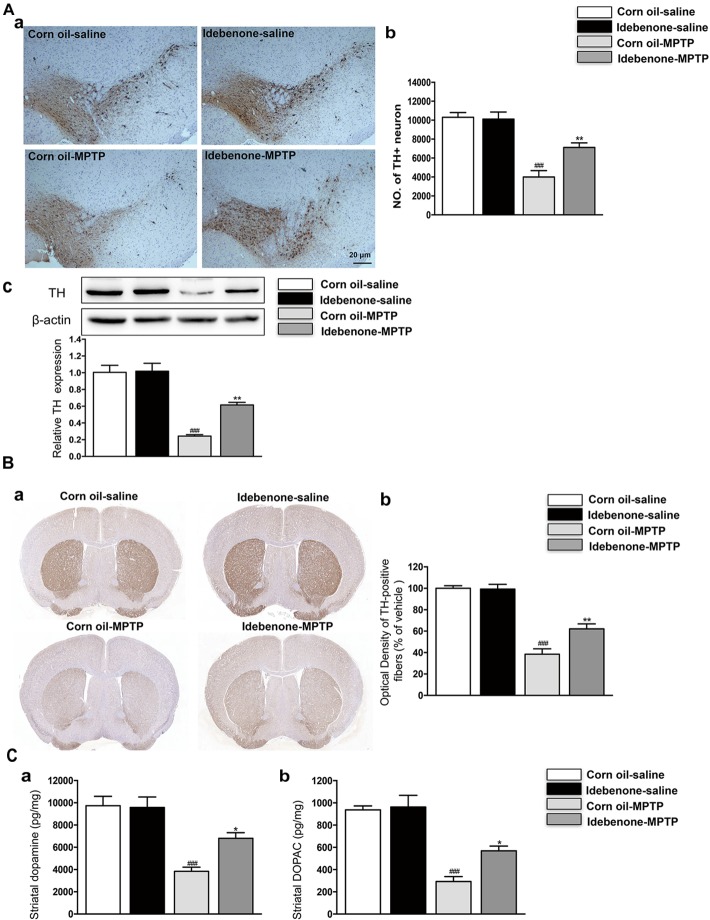
Idebenone treatments reduced MPTP-induced loss of TH and striatal dopamine and its metabolites, DOPAC. **(Aa)** Dopaminergic neurons were evaluated by immunohistochemical analysis of TH in the SN. Scale bar = 20 μm. **(b)** Quantification of the TH+ neuron in the SN. **(c)** The expression level of TH was analyzed by western blotting. **(Ba)** Optical density analysis for TH-positive fibers in the striatum. **(b)** Quantification of the TH in the striatum. **(C)** Striatal dopamine and its metabolites levels measured by HPLC (**a**: dopamine; **b**: DOPAC). Values are presented as the mean ± SEM. ^∗∗^*p* < 0.01: idebenone-MPTP vs. corn oil-MPTP; ^###^*p* < 0.001: corn oil-MPTP vs. corn oil-saline.

To determine whether idebenone in the PD model is beneficial or harmful to TH+ neurons in the SN, we compared the idebenone-MPTP group with its corn oil-MPTP group. The results showed that only 38% of TH+ neurons survived in the corn oil-MPTP group, while 66% of TH+ neurons survived in the idebenone-MPTP group (*p* < 0.01; Figures 6Aa,b). We also used western blotting to analyze the expression of TH in the SN. The results showed that expression of TH was downregulated in the SN of corn oil-MPTP-treated mice (*p* < 0.001; Figure 6Ac), whereas idebenone lessened the decline of TH in the SN, compared with corn oil-MPTP-treated mice (*p* < 0.01; Figure 6Ac).

In accordance with the previous reports (Zhu et al., 2018; Chen et al., 2019), MPTP-induced PD model decreased the TH-positive fibers and neurotransmitters, such as dopamine and its metabolites DOPAC in the striatum (*p* < 0.001; Figures 6B,C). Compared with corn oil-MPTP group, idebenone prevented TH positive fibers loss (*p* < 0.01; Figure 6B) and increased the dopamine and DOPAC levels (*p* < 0.05; Figure 6C). These results suggest a neuroprotective effect of idebenone against MPTP toxicity.

### Idebenone Treatment Inhibited the Inflammatory Response and Modulated Microglial Polarization in MPTP-Treated Mice

**FIGURE 7:**
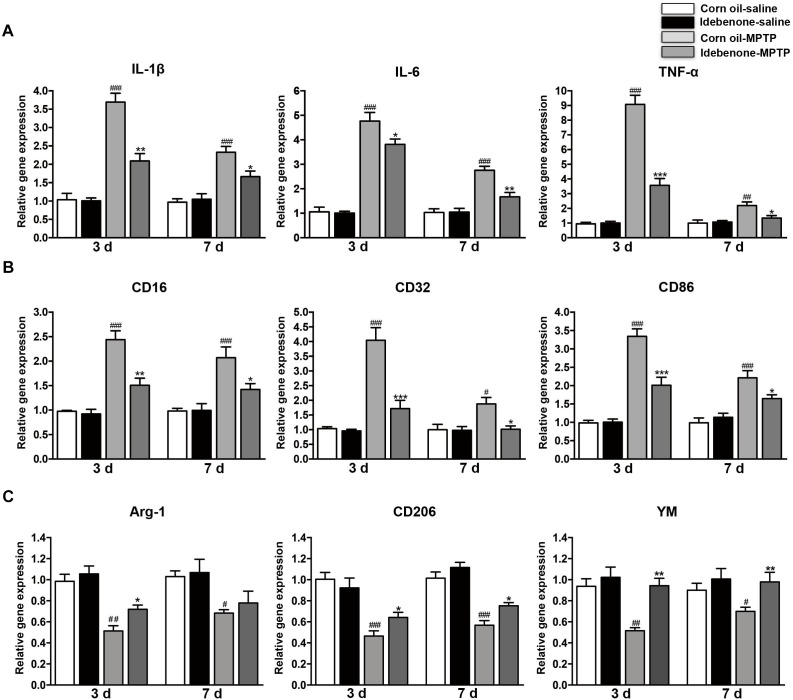
Idebenone treatments inhibited expression of proinflammatory cytokines and M1 markers and promoted M2 markers in the SN of PD mice. **(A)** The expression of proinflammatory factors (IL-1β, IL-6, and TNF-α) in the SN as determined by RT-qPCR. **(B)** The expression of M1 markers (CD16, CD32, and CD86) in the SN as determined by RT-qPCR. **(C)** The expression of M2 microglial markers (Arg-1, CD206, and YM) in the SN as determined by RT-qPCR. Values are presented as the mean ± SEM. ^∗^*p* < 0.05, ^∗∗^*p* < 0.01, ^∗∗∗^*p* < 0.001: idebenone-MPTP vs. corn oil-MPTP; ^#^*p* < 0.05, ^##^*p* < 0.01, ^###^*p* < 0.001: corn oil-MPTP vs. corn oil-saline.

**FIGURE 8:**
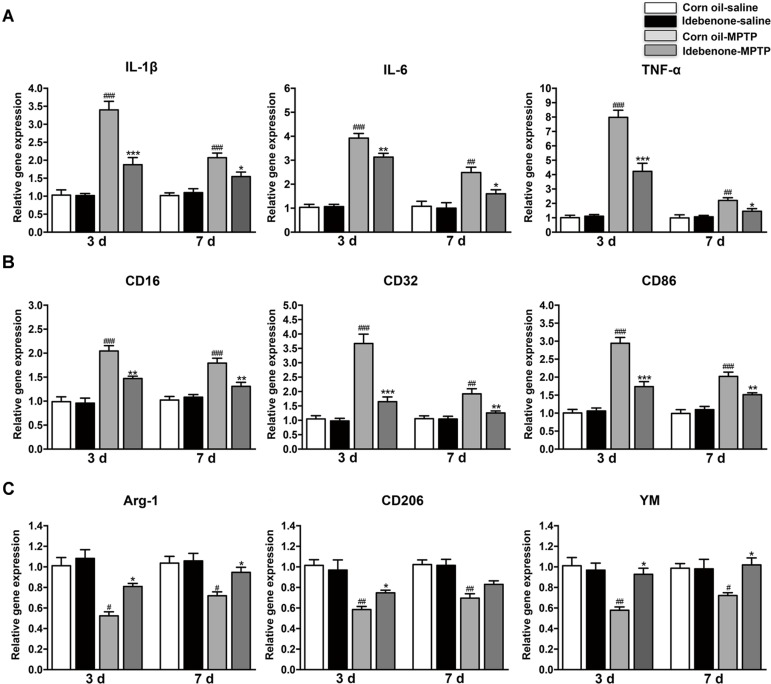
Idebenone treatments inhibited expression of proinflammatory cytokines and M1 markers and promoted M2 markers in the striatum of PD mice. **(A)** The expression of proinflammatory factors (IL-1β, IL-6, and TNF-α) in the striatum as determined by RT-qPCR. **(B)** The expression of M1 markers (CD16, CD32, and CD86) in the striatum as determined by RT-qPCR. **(C)** The expression of M2 microglial markers (Arg-1, CD206, and YM) in the striatum as determined by RT-qPCR. Values are presented as the mean ± SEM. ^∗^*p* < 0.05, ^∗∗^*p* < 0.01, ^∗∗∗^*p* < 0.001: idebenone-MPTP vs. corn oil-MPTP; ^#^*p* < 0.05, ^##^*p* < 0.01, ^###^*p* < 0.001: corn oil-MPTP vs. corn oil-saline.

To determine whether the protective effect of idebenone was related to anti-inflammatory effects and microglial phenotypic shift, we used RT-qPCR to detect proinflammatory cytokines and microglial phenotypic markers in the SN and in the striatum of mice sacrificed at 3 and 7 days after the last MPTP injection. The result indicated that the expression of proinflammatory cytokines (IL-1β, IL-6, and TNF-α) was significantly higher in the corn oil-MPTP treated mice than the corn oil-saline treated mice at 3 and 7 days after the last MPTP administration (*p* < 0.01; Figures 7A, 8A). In the idebenone-MPTP treated mice, idebenone inhibited the expression of IL-1β, IL-6, and TNF-α compared with the corn oil-MPTP treated mice (*p* < 0.05; Figures 7A, 8A).

To determine whether the anti-inflammatory effect of idebenone resulted from the regulation of microglial polarization in the SN and in the striatum of PD mice, we detected the expression of M1 and M2 markers by RT-qPCR. Increased mRNA expression of M1 markers (CD16, CD32, and CD86) and decreased mRNA expression of M2 markers (Arg-1, CD206, and YM-1) were observed at 3 and 7 days after the last MPTP administration (*p* < 0.05; Figures 7B,C, 8B,C). In animals pretreated with idebenone, M1 marker levels were decreased, and M2 markers increased gradually at 3 and 7 days after the last MPTP administration (*p* < 0.05; Figures 7B,C, 8B,C).

**FIGURE 9:**
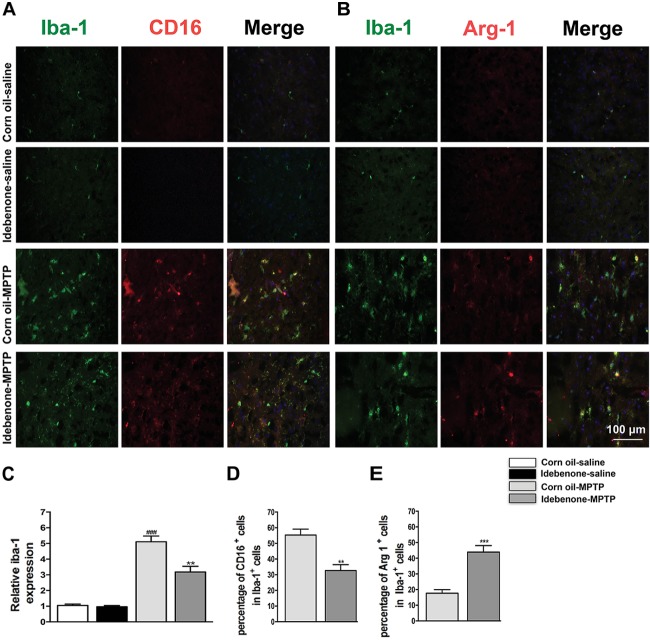
Idebenone reduces M1 phenotype and increased M2 phenotype in the SN of PD mice. **(A,B)** Immunofluorescence double staining for Iba-1 (green) with CD16 (red) or Arg-1 (red) in the SN at 3 days following MPTP injection. **(C)** The quantity of microglia in the SN is quantified by the intensity of Iba-1+ immunofluorescence. **(D,E)** Quantification of the percentage of CD16+/Iba-1+ and Arg-1+/Iba-1+ cells. Scale bar = 100 μm. Values are presented as the mean ± SEM. ^∗∗^*p* < 0.01, ^∗∗∗^*p* < 0.001: idebenone-MPTP vs. corn oil-MPTP; ^###^*p* < 0.001: corn oil-MPTP vs. corn oil-saline.

**FIGURE 10:**
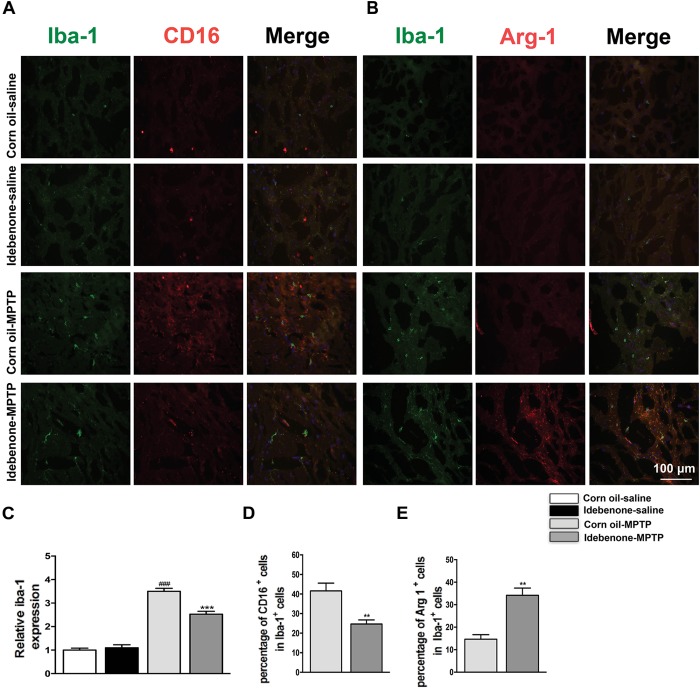
Idebenone reduces M1 phenotype and increased M2 phenotype in the striatum of PD mice. **(A,B)** Immunofluorescence double staining for Iba-1 (green) with CD16 (red) or Arg-1 (red) in the striatum at 3 d following MPTP injection. **(C)** The quantity of microglia in the striatum is quantified by the intensity of Iba-1+ immunofluorescence. **(D,E)** Quantification of the percentage of CD16+/Iba-1+ and Arg-1+/Iba-1+ cells. Scale bar = 100 μm. Values are presented as the mean ± SEM. ^∗∗^*p* < 0.01, ^∗∗∗^*p* < 0.001: idebenone-MPTP vs. corn oil-MPTP; ^###^*p* < 0.001: corn oil-MPTP vs. corn oil-saline.

The M1 and M2 signature genes are expressed not only in microglia but also in other central nervous system cells. The results of RT-qPCR reflected the changes of these genes in mixed cell types. To specifically evaluate the polarization state of microglia after MPTP injection, M1 marker (CD16) or M2 marker (Arg-1) were analyzed by double immunofluorescent staining with the microglia marker (Iba-1) in the SN and in the striatum. Consistent with the RT-qPCR results, expression of the M1 marker CD16 was lower in Iba-1+ microglia cells in idebenone-MPTP treated group compared with the corn oil-MPTP treated group at 3 days after MPTP injection (*p* < 0.01; Figures 9A,D, 10A,D). Expression of the M2 marker Arg-1 was higher in Iba-1+ microglia in idebenone-MPTP treated group compared with the corn oil-MPTP treated group (*p* < 0.01; Figures 9B,E, 10B,E).

We also evaluated the effect of idebenone on the numbers of microglia in the SN and in the striatum of MPTP mice. The percentage of Iba-1+ microglia in idebenone-MPTP treated group was obviously lower than that in corn oil-MPTP treated group (*p* < 0.01; Figures 9A–C, 10A–C). Our results suggested that idebenone could inhibit proliferation of the microglia in the SN and in the striatum.

### Idebenone Treatment Inhibited MPTP-Induced Activation of MAPK and NF-κB in the SN of PD Mice

**FIGURE 11:**
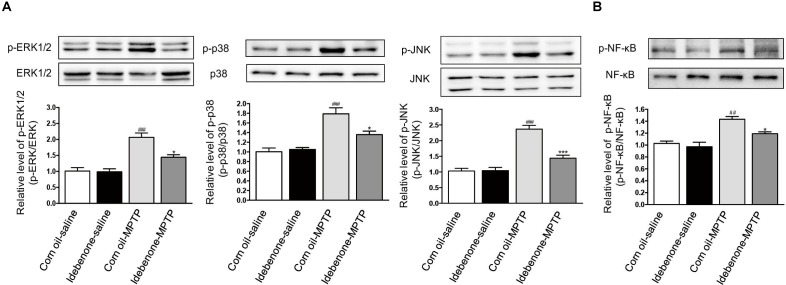
Idebenone inhibited the MPTP-induced activation of MAPKs and NF-κB in the SN of PD mice. **(A)** Expression levels of p-ERK, ERK, p-p38, p38, p-JNK, and JNK in the SN were analyzed by western blotting. **(B)** Expression levels of p-NF-κB and NF-κB in the SN were analyzed by western blotting. Values are presented as the mean ± standard error. ^∗^*p* < 0.05, ^∗∗∗^*p* < 0.001: idebenone-MPTP vs. corn oil-MPTP; ^##^*p* < 0.01, ^###^*p* < 0.001: corn oil-MPTP vs. corn oil-saline.

To confirm whether idebenone treatment suppressed activation of MAPK and NF-κB in the SN of PD mice, we tested the phosphorylation of MAPK/NF-κB in the mice. Our result showed that MPTP injection could induce the phosphorylation of p38, ERK, JNK, and NF-κB in the SN at 7 days after the last MPTP administration (*p* < 0.01, Figures 11A,B). Treatment of idebenone could inhibit the phosphorylation of ERK, p38, JNK, and NF-κB in the SN compared with the corn oil-MPTP mice (*p* < 0.05, Figures 11A,B).

## Discussion

Idebenone is a synthetic analog of the coenzyme Q10 (CoQ10), and its therapeutic potential has been reported in many diseases, including cerebrovascular disorders and neurodegenerative disorders (Nagaoka et al., 1989a,b). A growing number of studies suggest that idebenone protects against PD, and in agreement, we report that idebenone treatment not only ameliorated neurological dysfunction but also decreased the inflammatory response and modulated M1/M2 microglial polarization in LPS-activated BV2 microglial cells and MPTP-treated PD mice.

Accumulating evidence from human and animal studies suggests that the microglia-mediated neuroinflammatory response is a hallmark shared by PD (Xu et al., 2016). Microglia activation states are classified as the classically activated M1 state or the alternatively activated M2 state (Colton, 2009). LPS, an inducer of the M1 state, induces the release of several proinflammatory cytokines (Yang et al., 2017). These cytokines induce the expression of M1 markers (CD16, CD86) and elicit the progressive death of neurons in the SN (Tian et al., 2006). By contrast, CD206 and arg-1, which are markers of the M2 microglia, block the expression of proinflammatory cytokines and promote neuroprotection (Liu et al., 2013). The switch between the M1 state and M2 state has been regarded as a novel therapeutic strategy for neurodegenerative diseases (Yang et al., 2017).

Previously, some studies showed that idebenone had anti-inflammatory abilities. Lauro et al. (2016) reported that when complexed with HP-β-cyclodextrins, idebenone exerted a potent anti-inflammatory efficacy. Another observation of previous studies was that idebenone behaved similar to indomethacin and piroxicam—two typical anti-inflammatory agents (Civenni et al., 1999). In our study, we evaluated the anti-inflammatory effect of idebenone in LPS-activated BV2 microglial cells. Our results indicated that idebenone significantly attenuated the LPS-induced production of proinflammatory factors. Another finding from our research was that idebenone could inhibit LPS-stimulated M1 microglia activation and promote M2 microglia polarization *in vitro*. Furthermore, conditioned medium from the LPS-stimulated BV2 cells decreased SH-SY5Y cell viability, while pretreatment with idebenone significantly attenuated the effect of LPS. Our experiments indicated that LPS could induce proinflammatory cytokines released in BV2 cells, which may explain the reduction in SH-SY5Y cell survival in conditioned medium from activated BV2 cells.

To further investigate the potential mechanisms of inhibition of inflammatory factors release and modulation of microglia phenotypes by idebenone, different signal pathways activated by LPS were evaluated. NF-κB and MAPKs signaling pathways are significant regulators of LPS-induced expression of proinflammatory cytokines in microglia (Boje, 2004). Inhibition of these signaling pathways reduced the release of proinflammatory cytokines in LPS-activated BV2 cells (Yu et al., 2015). In our experiments, the phosphorylation of ERK, p38, JNK, and NF-κB were observed to occur within 30 min of LPS treatment and can be reduced by idebenone. These results demonstrated that idebenone inhibited LPS-induced activation of BV2 microglial cells by suppressing MAPKs and NF-κB activation.

For *in vivo* experiments, idebenone was treated at a dose of 100 mg/kg body weight. In previous studies, 100 mg/kg of idebenone improved neurological deficits and energy metabolism in animal with cerebral ischemia (Nagaoka et al., 1989a,b) and was shown to reduce age related neurodegeneration (Scavini et al., 1996). 100 mg/kg of idebenone was sufficient to attenuate neurological dysfunction following cerebral hypoxia in rats (Abdel Baky et al., 2010). Thus, in view of these research reporting administration of idebenone in mice, we considered 100 mg/kg idebenone to be a reasonable dose to test. Consistent with *in vitro* studies implicating the protective role of idebenone, we found that idebenone protected against MPTP-induced motor dysfunction and reduced the neuronal death in the SN and in the striatum. Idebenone also lessened the decline of dopamine levels and its metabolites in the striatum of MPTP-induced PD mice. As the dopamine level is a marker of dopaminergic synaptic function, these results indicate that idebenone exerts neuroprotective effects on MPTP-induced PD mice. This effect may be related to the inhibition of proinflammatory cytokines release, as evidenced by the decreased expression of IL-6, IL-1β, and TNF-α in the SN and in the striatum of MPTP-treated animals.

Conversion of M1 state to M2 state prevented death of TH+ neurons in the MPTP induced PD model (Zhao et al., 2015). Fasudil, a Rho kinase inhibitor, decreased the expression of M1 markers and increased the expression of M2 markers. Thus, fasudil promoted recovery of motor function and was neuroprotective (Zhao et al., 2015). In our study, idebenone suppressed the “proinflammatory” M1 phenotype, while enhancing the “anti-inflammatory” M2 microglia phenotype in the SN and striatum after MPTP administration. Our findings revealed that the anti-inflammatory effects and modulation of M1/M2 polarization by idebenone contributed to the survival of TH+ neurons in the MPTP-induced PD model. We attempted to identify the underlying mechanism that inhibits M1 polarization and promotes M2 polarization *in vivo*. Consistent with *in vitro* study, idebenone suppressed the phosphorylation of ERK, p38, JNK, and NF-κB in the SN of MPTP-treated animals. These data suggested that idebenone could inhibit M1 polarization and promotes M2 polarization by modulating the MAPKs and NF-κB signaling pathway *in vivo.*

Our work reveals a new insight into idebenone neuroprotection. Idebenone could attenuate the proinflammatory cytokines expression, and promote the phenotypic shift of microglia from the M1 to M2 phenotype in LPS-activated BV2 cells and the MPTP induced PD model. Mechanistically, these effects of idebenone may be mediated by inhibition of the MAPK and NK-κB pathway. Taken together, the present data suggested that the preventive potential of idebenone as a novel anti-inflammatory and microglia-modulating drug for neurodegenerative diseases associated with neuroinflammation, such as PD.

## Author Contributions

ZgL and AY designed the experiments. AY, ZhL, LS, XW, YZ, NW, JL, and YL performed the experiments. AY and ZhL analyzed the results. AY wrote the manuscript with contributions from ZgL. All authors read and approved the final manuscript.

## Conflict of Interest Statement

The authors declare that the research was conducted in the absence of any commercial or financial relationships that could be construed as a potential conflict of interest.

## Footnotes

1 http://www.cctcc.org

## Supplementary Material

FIGURE S1 Idebenone, LPS, idebenone plus LPS treatment did not induce cytotoxicity in SH-SY5Y cells. Idebenone, LPS, idebenone plus LPS were directly added to SH-SY5Y cells that had been seeded in 96-well plates for 24 h. CCK-8 assay kit was used to assess the changes in SH-SY5Y cells viability.
