# Supplementary figures and images for: Idebenone Alleviates Neuroinflammation and Modulates Microglial Polarization in LPS-Stimulated BV2 Cells and MPTP-Induced Parkinson’s Disease Mice
Source: Front Cell Neurosci. 2019 Jan 9;12:529. doi: 10.3389/fncel.2018.00529 (PMC6333870; doi:10.3389/fncel.2018.00529)

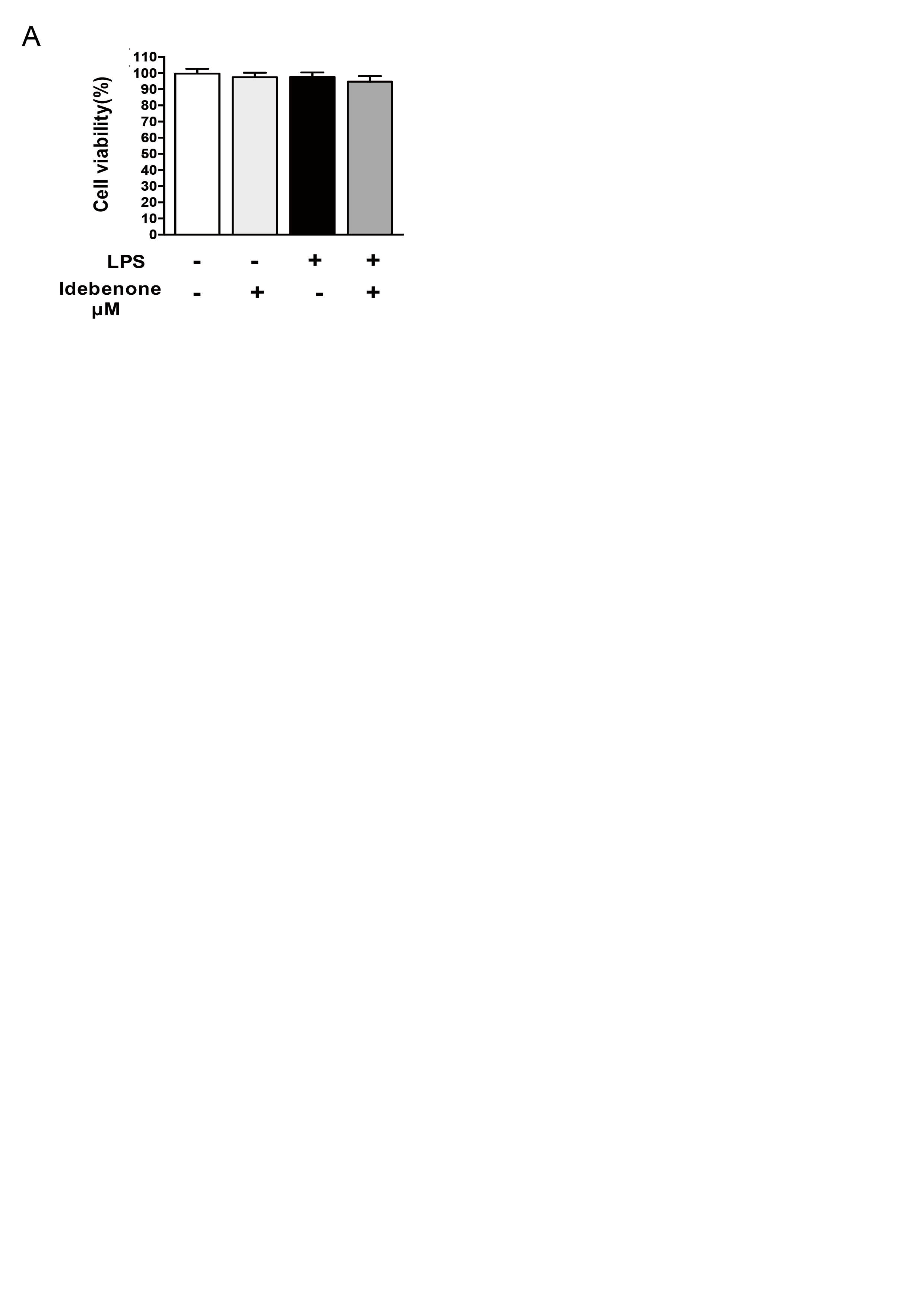

Supplement: FIGURE S1 — Idebenone, LPS, idebenone plus LPS treatment did not induce cytotoxicity in SH-SY5Y cells. Idebenone, LPS, idebenone plus LPS were directly added to SH-SY5Y cells that had been seeded in 96-well plates for 24 h. CCK-8 assay kit was used to assess the changes in SH-SY5Y cells viability. [file Image_1.JPEG]
